# Trachoma in Sudan: Risk Factors and Clinical Stages in Patients From Two Eye Care Hospitals, 2015

**DOI:** 10.7759/cureus.76394

**Published:** 2024-12-25

**Authors:** Rawan Hassan, Razan Hassan, Sagad Mohamed, Fadwa M Saad, Haidar A Mohamed

**Affiliations:** 1 Plastic Surgery, Derriford Hospital, Plymouth, GBR; 2 Community Medicine, Faculty of Medicine, University of Khartoum, Khartoum, SDN; 3 Anatomy, Faculty of Medicine, Alzaiem Alazhari University, Khartoum, SDN; 4 Pediatrics and Child Health, Faculty of Medicine, University of Khartoum, Khartoum, SDN

**Keywords:** clinical stages, hygiene, socioeconomic status, sudan, trachoma

## Abstract

Background

Trachoma is a leading infectious cause of blindness globally. While it has largely been eliminated in developed countries, it remains endemic in many developing regions. This study aimed to examine the clinical stages of trachoma and identify common sociodemographic and household characteristics associated with the disease among patients in Sudan.

Methods

We conducted a prospective, hospital-based descriptive cross-sectional study at Makah Eye Complex and Abdalfadeel-Almaz Hospital in Khartoum, Sudan. The study included trachoma patients of all age groups who visited these two eye care hospitals between March 1 and March 31, 2015. Data were gathered through clinical examinations, hospital records, and face-to-face interviews using a structured questionnaire.

Results

In this study, we identified 125 patients with trachoma, of whom 98 (78.4%) were over 50 years old. A majority, 70 (56%), tested positive for active trachoma; 51 (40.8%) had trachomatous trichiasis; and 70 (56%) exhibited trachoma follicles. Most patients were found to have low socioeconomic status (110, 88%), poor personal hygiene (55, 44%), and poor environmental hygiene (93, 74.4%).

Conclusions

Low socioeconomic status, poor personal hygiene, and inadequate environmental hygiene are prevalent sociodemographic and household characteristics among patients with trachoma. Despite significant progress toward disease elimination, trachoma continues to pose a public health challenge in Sudan.

## Introduction

Trachoma is a chronic cicatricial conjunctivitis caused by *Chlamydia trachomatis*, leading to progressive scarring of the eyelids and cornea [1]. It is the leading cause of preventable blindness worldwide, responsible for over 3% of all blindness cases. According to the WHO, trachoma is endemic in more than 50 countries, causing visual impairment in approximately 1.8 million people, of whom 0.5 million are irreversibly blind - about 1.4% of the global blind population [2,3]. Worldwide, around 232 million people live in trachoma-endemic areas [2,3].

Environmental risk factors, particularly poor hygiene, play a significant role in the disease’s transmission. Limited access to water, inadequate latrine use, and poor sanitation contribute to the spread of trachoma by creating breeding grounds for eye-seeking flies [4,5]. Personal risk factors include inadequate face and hand washing, irrespective of the distance to a water source [6]. The clinical manifestations of trachoma are categorized into “active” disease, typically seen in children, and “chronic” complications, such as trachomatous trichiasis and corneal opacification, which generally appear in late childhood and adulthood [7].

Trachoma has long been prevalent in parts of Sudan, with approximately 67,000 cases of TT in endemic areas. Sudan is one of the five countries that bear half of the global burden of active trachoma and one of the three countries that account for half of the global burden of TT [8]. However, there is a lack of studies in Sudan that examine the most common related factors and clinical stages of trachoma. Given this context, the current study aims to describe the common sociodemographic and household characteristics, as well as the clinical stages of trachoma observed in patients.

## Materials and methods

We conducted a prospective, hospital-based descriptive cross-sectional study at Makah Eye Complex and Abdalfadeel-Almaz Hospital in Khartoum State, Sudan, from March 1 to March 31, 2015. These two settings were selected because they are primary public healthcare facilities providing specialized ophthalmic services to a significant portion of the Khartoum State population.

We included all patients from all age groups with definitive clinical signs of trachoma, as per the WHO’s Trachoma Grading System. Sociodemographic and household characteristics were assessed through structured face-to-face interviews using a questionnaire. The study focused on the study population, demographic characteristics, and an evaluation of environmental and personal hygiene.

All patients who presented to the clinic were examined for trachoma before completing the questionnaire. If trachoma was suspected, they were referred to a consultant ophthalmologist for confirmation of the diagnosis and to receive the appropriate treatment.

Ethical considerations and consent

Prior to initiating the study, we obtained permission from the Faculty of Medicine at the University of Khartoum, as well as from the general directors of Makah Eye Complex and Abdalfadeel-Almaz Hospital in Khartoum, Sudan. Ethical approval was granted by the State Ministry of Health in Khartoum State, Sudan. Informed consent was obtained from each patient before their participation in the study.

## Results

Sociodemographic and household characteristics

The study included 125 patients with trachoma from two eye care hospitals: 80 patients (60.4%) from Makah Eye Complex and 45 patients (35.2%) from Abdalfadeel-Almaz Hospital. Among the participants, 68 (54.4%) were female and 57 (45.6%) were male. The majority, 98 (78.4%), were over 50 years of age. Most respondents, 77 (61.6%), were residents of Khartoum State, while 21 (16.8%) were from Gezira State. Additionally, 102 (81.6%) of the respondents did not have an infected person in their household (Table 1).

**Table 1:** Sociodemographic characteristics of the study population attending two eye care hospitals in Khartoum State, March 2015 (n = 125)

Variable	N	Percentage
Hospital		
Makkah Eye Complex	80	60.4%
Abdalfadeel-Almaz Hospital	45	35.2%
Gender		
Male	57	45.6%
Female	68	54.4%
Age		
Under 15 years old	1	0.8%
15-29 years old	7	5.6%
30-49 years old	19	15.2%
Over 50 years old	98	78.4%
Residence		
Khartoum State	77	61.6%
Gezira State	21	16.8%
Infected person in the family		
Present	102	81.6%
Absent	23	18.4%
Level of education		
Illiterate	66	52.8%
Khalwa	22	17.6%
Primary school	17	13.6%
Secondary school	11	8.8%
University graduate	8	6.4%
Postgraduate	1	0.8%
Monthly income		
Less than 1,000 SDG	36	28.8%
1,000-2,000 SDG	9	7.2%
More than 2,000 SDG	3	2.4%
Unemployed	76	60.8%

Regarding drinking water, most respondents, 98 (78.4%), had a pipe inside their house, and 10 (8%) had a pipe outside the house as their water source. More than half of the respondents, 74 (59.2%), owned animals, with 65 (52.2%) keeping them inside their homes. The majority of respondents, 92 (74.2%), used pit latrines as their toilet type, while three (2.4%) had no toilet facilities. Most respondents, 86 (68.8%), washed their faces two to five times per day, and six (4.8%) never washed their faces. Regarding handwashing, the majority, 68 (54.4%), washed their hands two to five times per day (Table 2).

**Table 2:** Environmental factors and personal hygiene among patients with trachoma attending two eye care hospitals in Khartoum State, March 2015 (n = 125)

Variable	N	Percentage
Drinking water source		
House pipe	89	78.4%
External pipe	1	0.8%
Wells	6	4.8%
Rivers	2	1.6%
Place of animal breeding (if present)		
Inside the house	66	86.8%
Inside the residential block	10	13.2%
Type of toilet		
Flush toilet	29	23.4%
Pit latrine	92	74.2%
No toilet	4	2.4%
Face washing per day		
Once	8	6.4%
Two to five times	86	68.8%
More than five times	25	20%
Never	6	4.8%
Handwashing per day		
Once	4	3.2%
Two to five times	68	54.4%
More than five times	52	41.6%
Never	1	0.8%

Clinical stages of trachoma

All patients were examined by an ophthalmologist using a slit lamp, and the clinical signs of trachoma were graded according to the WHO’s Trachoma Grading System. The majority of respondents, 70 (56%), were found to have trachoma follicles, while 51 (40.8%) had trachomatous trichiasis (Table 3).

**Table 3:** Clinical stages of trachoma among patients attending two eye care hospitals in Khartoum State, March 2015 (n = 125)

Clinical stage	N	Percentage
Trachoma follicles	70	56%
Trachoma scarring	2	1.6%
Trachoma trichiasis	51	40.8%
Corneal opacification	2	1.6%

## Discussion

Most of the patients in this study were females over 50 years of age, with low socioeconomic status and poor personal and environmental hygiene. Unlike a study conducted in a Gambian village, which found trachoma primarily in children, the majority of patients in this case series were over 50 years old [9]. The proportion of female patients exceeded that of males, a predictable outcome stemming from mothers’ roles as caregivers and their close proximity to the trachoma reservoir - children under 15 years old - putting them at higher risk of contracting the disease. This finding aligns with previous studies [10].

We observed that most of the patients in this study initially presented with complaints unrelated to trachoma, leading to an accidental diagnosis. This is understandable, as doctors often consider trachoma to be uncommon in today’s world, particularly in Khartoum State. This misconception affects the accurate determination of the disease’s true prevalence.

In terms of socioeconomic status, the majority of respondents were in a disadvantaged position. Poor socioeconomic status is associated with poor housing and overcrowding, which increases the risk of developing and transmitting the infection [7,11]. Furthermore, 18.4% of respondents reported having a family member with trachoma. Studies have shown the clustering nature of the disease at the community, household, and bedroom levels, reflecting the dynamics of transmission among family members with prolonged close contact [12-14]. However, patients may be unaware if someone in their family is carrying trachoma asymptomatically, potentially underestimating the disease burden and delaying treatment-seeking behavior.

Regarding personal hygiene, the majority of respondents exhibited inadequate facial cleanliness and handwashing practices. Numerous studies [6,15,16] have addressed facial cleanliness, a key component of the WHO-SAFE strategy. A “dirty face” is defined by the presence of ocular and/or nasal discharge [6].

Most respondents also had poor environmental hygiene. Several studies have reported that as the distance to the water supply increases, the prevalence of trachoma rises [17]. Other studies have demonstrated that the quantity of water used for face washing is more important than the distance to water [18]. More than half of the respondents owned pets, with 86.8% keeping them indoors. Animal feces serve as a suitable breeding ground for flies, which are known vectors for *C. trachomatis*. This has been confirmed by numerous other studies [19]. Additionally, the majority of respondents (73.6%) used pit latrines inside their homes, which also creates an ideal environment for flies [4-6].

This article was published as a preprint on the Research Square server on July 22, 2020 [20].

Limitations

The study was conducted at the two main eye care hospitals in Khartoum, but it did not include patients who presented to other hospitals or asymptomatic individuals who did not seek medical attention, potentially underrepresenting the true extent of trachoma. Furthermore, the fact that the majority of patients were residents of Khartoum may be attributed to the study’s focus on hospitals located within Khartoum itself.

## Conclusions

Low socioeconomic status and poor personal and environmental hygiene are common sociodemographic and household characteristics among patients with trachoma. A lack of awareness may contribute to the underestimation of the true magnitude of the disease. Despite progress toward its elimination, trachoma remains a significant public health problem in Sudan.
